# Development of Cerium Oxide/Corncob Nanocomposite: A Cost-Effective and Eco-Friendly Adsorbent for the Removal of Heavy Metals

**DOI:** 10.3390/polym13244464

**Published:** 2021-12-20

**Authors:** Sidra Gran, Rukhsanda Aziz, Muhammad Tariq Rafiq, Maryam Abbasi, Abdul Qayyum, Ashraf Y. Elnaggar, Hussein H. Elganzory, Zeinhom M. El-Bahy, Enas E. Hussein

**Affiliations:** 1 Department of Environmental Science, International Islamic University, Islamabad 44000, Pakistan; sidragran@gmail.com (S.G.); maryam.abbasi@iiu.edu.pk (M.A.); 2 Centre for Interdisciplinary Research in Basic Sciences, International Islamic University, Islamabad 44000, Pakistan; tariq.rafiq@iiu.edu.pk; 3 Department of Agronomy, The University of Haripur, Haripur 22620, Pakistan; 4 Department of Food Nutrition Science, College of Science, Taif University, Taif 21944, Saudi Arabia; aynaggar@tu.edu.sa; 5 Department of Chemistry, College of Science, Qassim University, Buraidah 51452, Saudi Arabia; hhsien@qu.edu.sa; 6 Department of Chemistry, Faculty of Science, Al-Azhar University, Cairo 11884, Egypt; zeinelbahy@azhar.edu.eg; 7 National Water Research Center, Shubra El-Kheima 13411, Egypt; enas_el-sayed@nwrc.gov.eg

**Keywords:** corncob, cerium oxide, nanocomposite, adsorption, heavy metals

## Abstract

This research aims to assess the efficiency of the synthesized corncob as a cost-effective and eco-friendly adsorbent for the removal of heavy metals. Therefore, to carry out the intended research project, initially, the corncob was doped with nanoparticles to increase its efficiency or adsorption capacity. The prepared adsorbent was evaluated for the adsorption of cadmium (Cd) and chromium (Cr) from aqueous media with the batch experiment method. Factors that affect the adsorption process are pH, initial concentration, contact time and adsorbent dose. The analysis of Cd and Cr was performed by using atomic absorption spectrometry (AAS), while the characterization of the adsorbent was performed using Fourier transform infrared spectroscopy (FTIR), X-ray diffraction (XRD), and scanning electron microscopy (SEM). The results showed that there is a significant difference before and after corncob activation and doping with CeO_2_ nanoparticles. The maximum removal for both Cd and Cr was at a basic pH with a contact time of 60 min at 120 rpm, which is 95% for Cd and 88% for Cr, respectively. To analyze the experimental data, a pseudo-first-order kinetic model, pseudo-second-order kinetic model, and intra-particle diffusion model were used. The kinetic adsorption studies confirmed that the experimental data were best fitted with the pseudo-second-order kinetic model (R^2^ = 0.989) and intra-particle diffusion model (R^2^ = 0.979). This work demonstrates that the cerium oxide/corncob nanocomposite is an inexpensive and environmentally friendly adsorbent for the removal of Cd and Cr from wastewater.

## 1. Introduction

Water is a very precious and essential natural resource in the world. As time passes, the quality of water is deteriorating mostly due to human activities, demographic expansion, industrialization urban sprawl, and unrestrained consumption of natural resources [1]. Therefore, improving and preserving water quality is essential.

The metals/metalloids that have an atomic number of more than 20 and density higher than 5 g/cm^3^ are known as heavy metals [2]. At relatively low concentrations, these metals are toxic or even poisonous. Due to the non-degradable nature of heavy metals, they are common contaminants of water and soil and cause risks to the ecosystems; however, toxic heavy metals in water create serious health and environmental hazards [3]. The main sources of Pb, Cd, and Cr in wastewater are paints, fertilizers, petrol, pigments, electroplating, plastic industries cables, steel, alloys, metal polishing, and photographic material [4,5]. Due to their high toxicity and mobility in the environment, the removal of Cd and Cr from aqueous media should be of great concern.

Contact with heavy metals, even at very low concentrations, can be harmful to many parts of the human body. Hence, the removal of heavy metals from solutions is extremely significant [6]. Several health problems can be caused by water polluted with metallic discharge as [7] stated that Cd causes osteomalacia and kidney tubular destruction. Therefore, it is of great importance to remove heavy metals from the aquatic environment by using cost-effective and energy efficient adsorbents.

Several methods and materials are used to depollute water loaded with heavy metals [8,9]. They include chemical precipitation, ion exchange, adsorption, membrane filtration, reverse osmosis, solvent extraction, and electrochemical treatment [10]. Some of these techniques, however, have drawbacks, including a high maintenance and operational cost, complex procedures involved in these treatments, and the generation of toxic sludge [11]. These technologies, nonetheless, have proven to be either inefficient or costly. 

Furthermore, nanomaterials have some special properties, such as their size, adsorbent capacity, macro quantum tunnel effect, and quantum effect [12]. Because of their reduced sizes, nanomaterials have high surface-to-volume ratios, which make them highly reactive with discrete characteristics [13]. Hence, such variable properties are related to the remarkable reactivity and adsorption capacity of these nanomaterials. 

CeO_2_ is a lanthanide, a rare Earth metal oxide that has a face-centered cubic structure [14]. In recent years, CeO_2_ has attracted much attention due to its exceptional physical and chemical properties. The morphologies, microstructures, characterizations, and theoretical studies of cerium-based nanomaterials have been widely examined in the past few years with some extraordinarily encouraging outcomes [13]. 

Agricultural waste materials have received great attention and are considered alternative adsorbents mainly due to their easy availability, cost-effectiveness, and high efficiency of a different kind of heavy metal ions adsorption [15,16]. Hence, the availability and abundance of agro-wastes make them a good source of raw materials for natural adsorbents. Agro-waste materials include a variety of functional groups, such as lignin, lipids, hemicelluloses, proteins, water, hydrocarbons, starches, and simple sugars. Specifically, agro-materials have cellulose and exhibit a high sorption capability for a variety of contaminants [17]. Such waste material can be used as cheap adsorbents, which would have double of advantages, especially in environmental and economic terms.

Corn is a common cereal crop grown in various parts of the world [18]. The corn milling process produces corncobs that are very voluminous and a costless agricultural waste. The numerous polymeric materials of corn cob contain many functional groups [19]. Corncobs are very rich in cellulose and hemicelluloses and are comprised 44% of cellulose. Cellulose has hydrophilic characteristics due to the presence of hydroxyl groups in each polymer unit [20]. Corncob can be used as an adsorbent for the removal of heavy metals due to the presence of lignin, cellulose, pectin, and hemicelluloses, which possess functional groups as binding sides for metal ions [21]. The corncobs are considered carbonaceous materials, but a large amount of them end up in landfill as waste.

This study aims to examine the adsorption behavior of selected heavy metals on a CeO_2_/corncob nanocomposite from aqueous solutions and thus the possibility to remove Cd and Cr heavy metals from wastewater. Corncobs are produced in large amounts in Pakistan and, instead of being dumped in landfills, they can be used as a green solution for the removal of Cd and Cr from wastewater. 

The purposes of this research are: (i) the synthesis and characterization of the dried corncob nanocomposite; (ii) the application of the nanocomposite for the efficient removal of heavy metals from aqueous media; and (iii) to study the effect of pH, initial concentration, dose, and contact time required for bio-sorption. To our knowledge, no work has been reported on a cerium oxide corncob nanocomposite as an adsorbent for the removal of heavy metals from wastewater.

## 2. Materials and Methods

### 2.1. Chemicals

All chemicals used in the present work were of analytical purity. The chemicals used were potassium chromate, cadmium chloride, sodium hydroxide, hydrochloric acid, ethanol, and acetone. The chemicals were purchased from Prime Chemicals Corporation (Pvt), Ltd. (Karachi, Pakistan).

#### 2.1.1. Preparation of Corncob

Maize corncobs were collected from the local market of Swat KPK, and external materials, such as dirt and sand, were removed from corncobs. They were washed with tap water and then the sample materials were sun-dried for 8–10 days. The sun-dried samples were cut into small pieces and then crushed into a fine powder using a grinder. The crushed fine powder was washed with distilled water. The procedure of washing was repeated until the corncob crushed powder was colorless and then it was dried at 80 °C for 8 h.

#### 2.1.2. Activation of the Corncob

After properly drying, 10 g of pre-activated corncob and 100 mL 2 M NaOH solution were placed in a 500 mL reaction kettle and heated at 80 °C with 200 rpm for 7 h. the corncob sample was treated with NaOH to remove impurities, which increased the adsorption capacity of the corncob, as Hoyos-Sánchez et al. [22] reported that washing rice husks with NaOH increased their adsorption capacity. According to Kumar and Bandyopadhyay [23], washing the adsorbent with NaOH also removes the impurities that can disturb the adsorption process.

Further, the obtained corncob sample was filtered out overnight and the material was washed until the filtrate became colorless and neutral. Approximately after 12 to 14 washings, it became colorless and neutral, and was then treated in hydrochloric acid (2 M) underwater bath at 80 °C for 25 min to eliminate the remaining alkali metal elements in the material. Finally, this prepared activated corncob was washed until the filtrate became neutral and dried in an oven overnight at 60 °C.

#### 2.1.3. CeO_2_ Nanoparticle

The co-precipitation method was used for the preparation of cerium oxide nanoparticles as reported by [24].

#### 2.1.4. Preparation of Nanocomposites of Cerium Oxide and Activated Corncob

A total of 5 g of activated corncob powder was mixed with 1 g of CeO_2_ nanoparticles, disseminated in 30 mL of distilled water, and stirred through a magnetic stirrer for half an hour. The prepared sample was moved to a 50 mL reactor and heated at 80 °C for 12 h, washed two times with ethanol and distilled water, followed by drying in an oven at 60 °C. This product was marked as a CeO_2_/Corncob nanocomposite.

#### 2.1.5. Calcination

CeO_2_/corncob nanocomposite was placed in a muffle furnace at 500 °C for 2 h for calcination. This fine powder was then placed in a desiccator and ready to use as CeO_2_/corncob nanocomposite.

### 2.2. Characterization

Scanning electron microscopy was carried out at different magnifications to study the surface morphology of the corncob and CeO_2_/corncob nanocomposite by using KYKEM6900 (Eindhoven, The Netherlands). SEM was carried out at voltage 30 KV, current 2.7 A, and distance 10–15 mm. The XRD patterns of corncob and CeO_2_/corncob was obtained by using D8 Advance X-ray powder diffraction microscopy. The functional groups on adsorbent surfaces were identified by the FTIR technique (PerkinElmer, Waltham, MA, USA) at an infrared (IR) range of 4500–500 cm^−1^; to conduct the FTIR spectroscopy, approximately 0.6 mg of the sample was used. 

#### Batch Adsorption Experiment

CeO_2_/corncob nanocomposite was applied as an adsorbent for the removal of Cd and Cr. The stock solution of metal salts was prepared in distilled water and dilutions were made to prepare the working solutions. The stock solution of Cd and Cr was prepared in 100 mL concentration by using CdCl_2_ and K_2_CrO_4_, NaOH and HCl were used for the adjustment of the pH. The batch experiment was carried out under variable parameters of the initial concentration of metal ions, pH, and dose of the adsorbent at a steady shaking speed of 100 rpm and contact time 60 min.

By adding 0.01 M HCl or 0.01 M NaOH, the pH of the solution was settled; the pH of the solution was measured using a pH meter (inoLab). The concentration of the Cd and Cr in the solution was measured with the help of an atomic absorption spectrophotometer (AA-7000 SHIMADZU, Eindhoven, The Netherlands).

## 3. Results and Discussion

### 3.1. Scanning Electron Microscopy (SEM) Analysis

**Figure 1:**
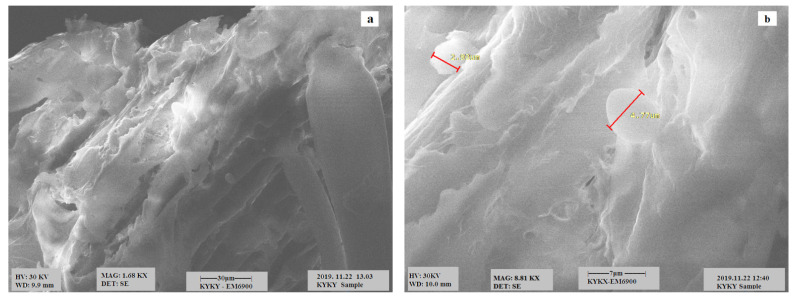
SEM images of the corncob under magnification of 1.68K× (**a**) and 8.81K× (**b**).

**Figure 2:**
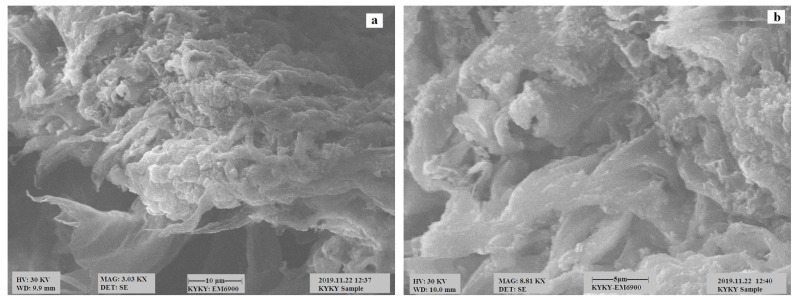
SEM images of the CeO_2_/corncob nanocomposite under magnification of 3.03K× (**a**) and 8.81K× (**b**).

SEM micrographs of the samples of corncob are depicted in Figure 1a,b. It is evident from the image that the corncob is a smooth material without any pores. Figure 2a,b represents the surface morphology of the CeO_2_/corncob nanocomposite, possessing a large number of tiny pores; as Rangabhashiyam et al. [25] stated, for an efficient biosorption, an internal surface and pores are necessary.

### 3.2. Fourier Transform Infrared Spectroscopy (FTIR)

**Figure 3:**
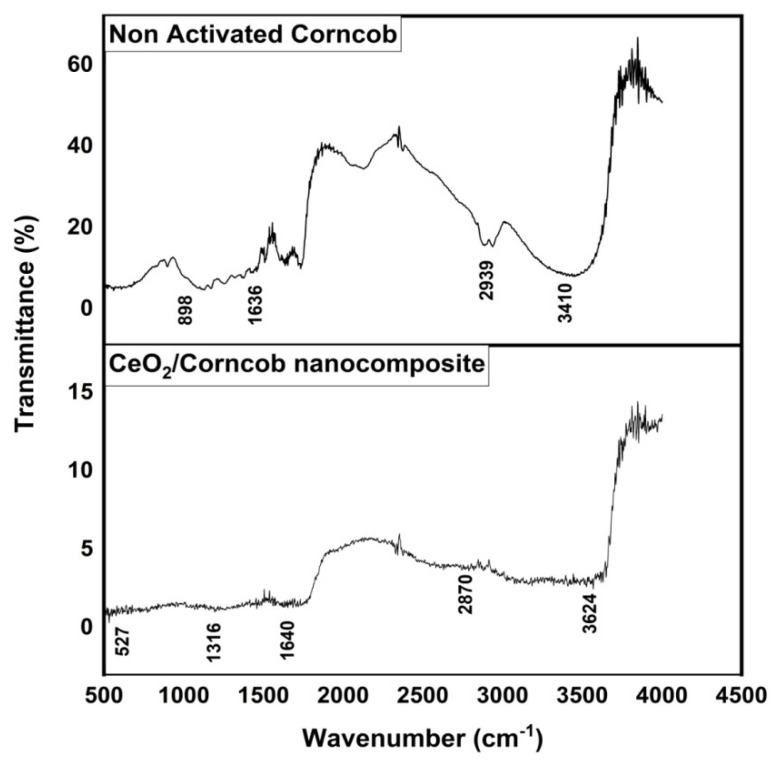
FTIR spectra of the corncob and CeO_2_/corncob nanocomposite.

Functional groups on adsorbent surfaces can be effectively identified by the FTIR technique, which is capable of adsorbing pollutant ions [26]. Figure 3 indicates the FTIR spectrum of the corncob and CeO_2_/corncob nanocomposite, and an examination of the surface of the adsorbent before and after the nanocomposite formation showed the functional groups involved in the activation and modification of the surface area of the adsorbent.

A broadband in the region of 3410 cm^−1^ indicates the inner and outer –OH stretching vibrations. The peak obtained at 2939 cm^−1^ is due to the C–H stretching of aliphatic carbon. The amount of 1636 cm^−1^ may represent OH bending of water or may be attributed to the COO symmetric stretching of hemicellulose, lignin, and amino groups [27]. The presence of the above-mentioned functional groups matches with the previous literature [28]. In comparison, it can be observed from Figure 3 that there is a significant difference in the FTIR spectra of the corncob and CeO_2_/corncob nanocomposite. The peaks witnessed at 3410 cm^−1^, 2939 cm^−1^, and 898 cm^−1^ are diminished in CeO_2_/corncob spectra, which shows that –OH and C-H linkages break due to the presence of CeO_2_ in the structure. This shows the successful formation of CeO_2_/corncob nanocomposite.

### 3.3. X-ray Diffraction (XRD) Analysis

**Figure 4:**
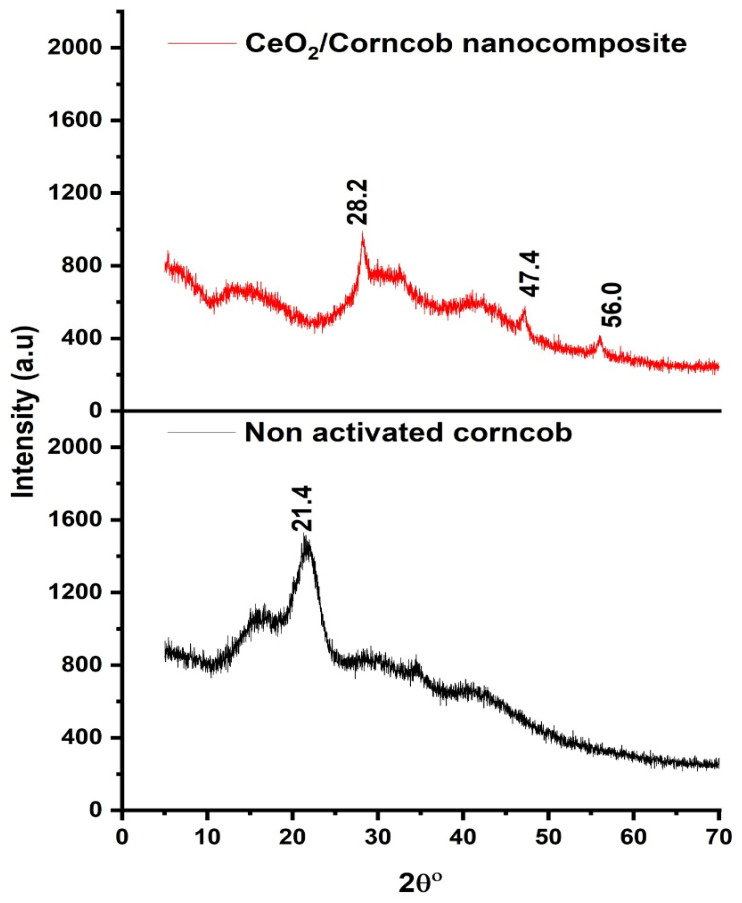
XRD spectra of the corncob and CeO_2_/corncob nanocomposite.

The XRD patterns of corncob and CeO_2_/corncob are represented in Figure 4. The XRD graphs demonstrate that there is a significant change in the structure of corncobs before and after presence of CeO_2_. The bulk of the X-ray signal originated from the corncob substrate in 2θ = 21.4° matched well with the previous literature reported by [29]. The XRD of the CeO_2_/corncob nanocomposite shows three diffraction peaks at 2θ angle = 28.2°, 47.4°, and 56.0°. The absence of sharp peaks confirms the amorphous nature of the CeO_2_/corncob nanocomposite.

### 3.4. Batch Adsorption Experiment

Batch experiments were carried out to determine the adsorption capacity of the CeO_2_/corncob nanocomposite.

#### 3.4.1. Effect of the pH on the Removal of Cd and Cr

**Figure 5:**
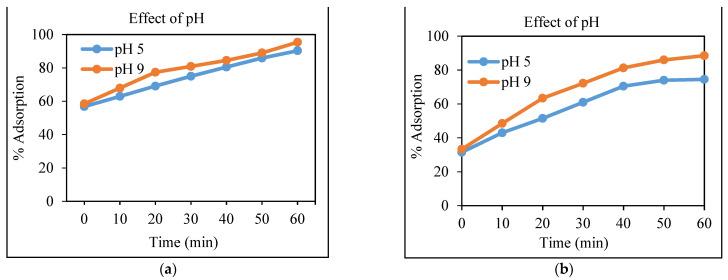
Removal (in percentage) of Cd (**a**) and Cr (**b**) using the CeO_2_/corncob nanocomposite as a function of pH (at an optimum dose and concentration).

A key controlling parameter in the bio-sorption process of toxic substances from an aqueous solution is the pH because, during the reaction, it affects the concentration of the counter ions on the functional groups of the adsorbent, the degree of ionization of the adsorbate, and the solubility of the ions [30]. To examine the effect of the pH on the adsorption of Cd and Cr, the experiment was carried out at two varying pH, including mildly acidic (pH 5) and mildly alkaline (pH 9). According to the results given in Figure 5a,b, the adsorption process is quite rapid in both acidic and basic pH, but, as the pH changed from 5 to 9, the percentage removal of Cd and Cr increased. The maximum percentage removal at pH 9 was found to be 95% and 88% for Cd and Cr, respectively, as Okorie et al. [28] reported that the percentage removal of Pb(II) and Cd(II) on the adsorbents increased with an increase in pH. As the pH increased from 2 to 8, the percentage removal of the Pb(II) increased from 14% to 99%; similarly, the adsorption of the Cd(II) increased from 13% to 86% as the pH increased from 2 to 8. As the pH value increased, the H+ ions in solution decreased, while H- ions increased; then, the competition between the metals and H+ ions for the active sites of the adsorbents also reduces, which leads to an increase in percentage removal of the adsorbent [31]. From this study, we know that pH significantly affects the adsorption of Cd and Cr by the corncob nanocomposite. The results demonstrate that a maximum removal of chromium was found at pH 9.

#### 3.4.2. Effect of the Adsorbent Dose

**Figure 6:**
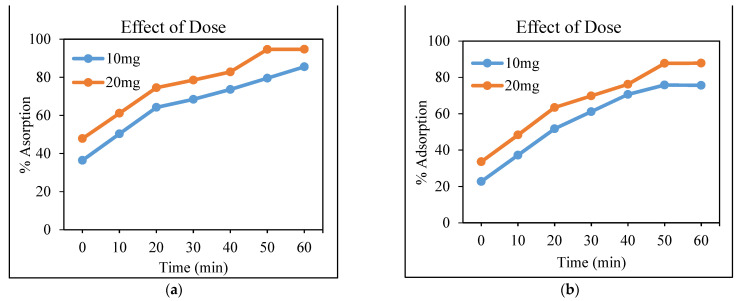
Removal (in percentage) of Cd (**a**) and Cr (**b**) using the CeO_2_/corncob nanocomposite as a function of adsorbent dose.

The adsorbent dose plays an important role to determine the capacity of an adsorbent for the concentration of the adsorbate. To study the effect of the adsorbent dose, varied doses were used for the removal of Cr and Cd. According to the results presented in Figure 6a,b, it is demonstrated that, as the adsorbent dose increases, the percentage removal of the adsorbate also increases. The same findings have been reported by Garg et al. [19]: the percentage adsorption of the adsorbate increased with an increase in the dose of the adsorbent. As the dose of the adsorbent increases, it certainly enhances the number of adsorption sites and thus increases the surface area of the adsorbent [32]. It can be concluded from the results that the optimum dose is 20 mg because the maximum removal is 95% for Cd and 88% for Cr. 

#### 3.4.3. Effect of Concentration

**Figure 7:**
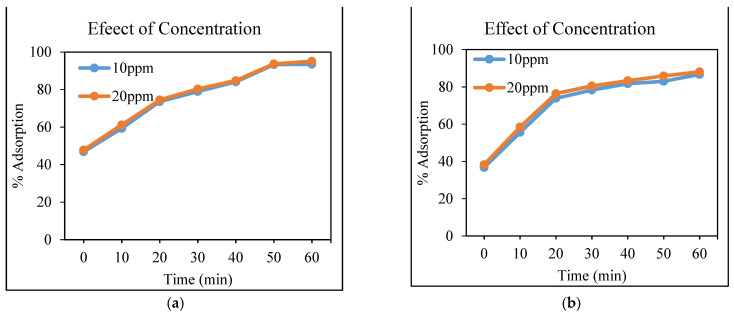
Removal (in percentage) of Cd (**a**) and Cr (**b**) using the CeO_2_/corncob nanocomposite as a function of initial concentration (at optimum dose).

The purpose of this experiment was to investigate the effect of initial concentration on the adsorption of heavy metals (Cd and Cr) on the CeO_2_/corncob nanocomposite. In this study, to investigate the effect of the adsorbate’s initial concentration, two adsorbate concentrations were chosen, namely 10 ppm and 20 ppm. According to the experimental results given in Figure 7, it can be concluded that the adsorption process increases with an increase in the adsorbate’s concentration, which mainly occurs because of the increase in the number of adsorbent sites and of the adsorbate particles. The adsorption capability increased as the concentration of adsorbate increased. That is because, at low concentrations, the ratio of the initial number of heavy metal molecules to the available surface area is low. At a high concentration, the ratio is higher because there are fewer available sites, so the removal rate of heavy metals depends upon the concentration. The same trend has been observed in the adsorption of carbaryl treated with eggshells [33]. Our results are also in agreement with [34], which explains that the percentage removal of Cr, Cd, and Cu metal ions was increased with an increase in the concentration.

#### 3.4.4. Effect of Contact Time on the Removal of Cd and Cr

**Figure 8:**
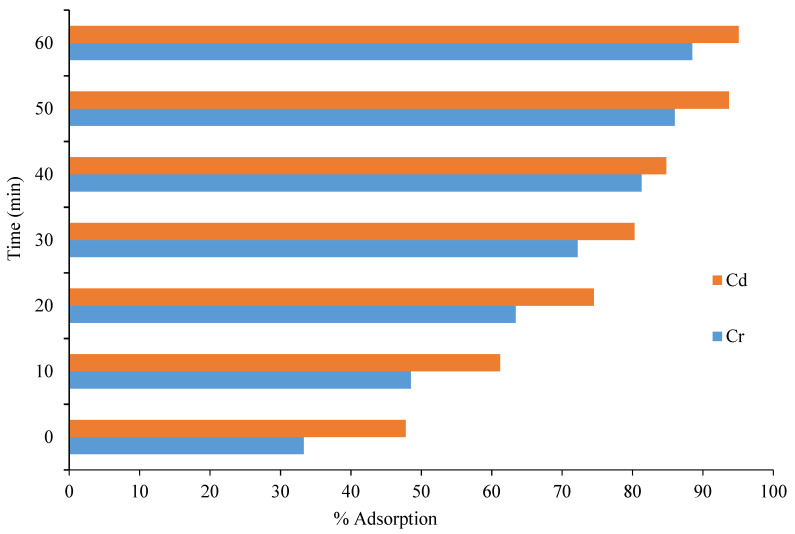
Removal (in percentage) of Cd and Cr using the CeO_2_/corncob nanocomposite as a function of time.

The effect of contact time on the adsorption of Cd and Cr was investigated to determine the maximum time taken to attain the equilibrium. The effect of contact time was examined over the range of 0–60 min. It can be observed from the results, shown in Figure 8, that, as the contact time increased, the percentage removal also increased, which might be due to the availability of active sites in the adsorbent. The removal of Cd increased from 47.8% to 95.5% as the time increased from 0 to 60 min; the removal of Cr increased from 33.3% to 88.5% as the time increased from 0 to 60 min, at a shaking speed of 120 rpm. Overall, the percentage removal for both Cd and Cr increased as the contact time increased from 0 to 60 min.

### 3.5. Adsorption Kinetics

In this research, adoption kinetic models were used to evaluate how adsorbates interacted with the adsorbent. To study the mechanism of the adsorption process, the experimental data were evaluated with a pseudo-first order, pseudo-second order kinetic model, and Morris intra-particle diffusion model [35].

In 1898, Lagergren proposed the pseudo-first-order model that is expressed by the following equation:

*dqt*/*dt* = *k*1(*qe* − *qt*) (1)

where *qt* and *qe* are the amount of solute adsorbed at time *t* (min) and at equilibrium, respectively, and *k*1 (min^−1^) represents the adsorption rate constant. The integrated form of this equation is represented by the following Equation (2):

*log* = (*qe* − *qt*) = *log qt* − *k*1*t* (2)

**Figure 9:**
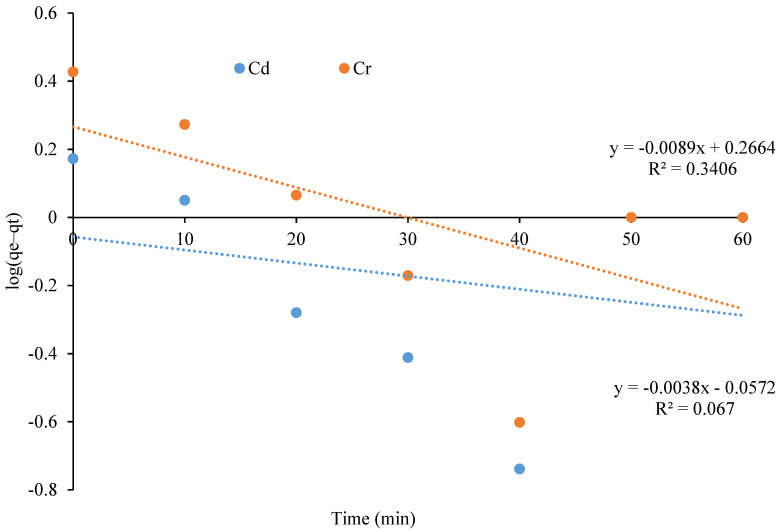
Pseudo-first-order kinetic plot of metal adsorption on the CeO_2_/corncob nanocomposite.

Figure 9 represents the plot of log (*q_e_* − *q_t_*) versus *t*. The regression values show that the fitness of the model is not applicable for Cd and Cr. The results obtained by [34] also revealed that the value of R^2^ for the pseudo-first-order model was poor, which demonstrated an unfit model for the adsorption of heavy metals.

**Figure 10:**
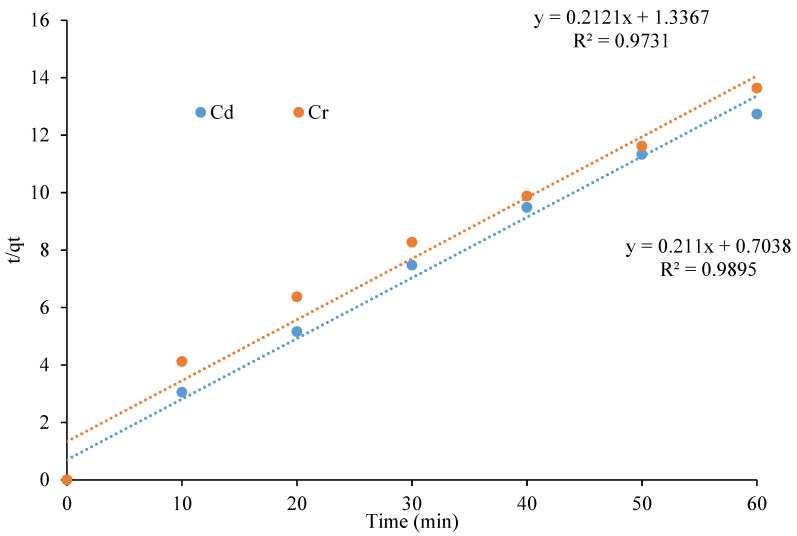
Pseudo-second-order kinetic plot of metal adsorption on the CeO_2_/corncob nanocomposite.

The pseudo-second-order model was first applied by Ho et al. in 1996 and is represented by the following equation:

*t*/*qt* = 1/*k*2*qe*^2^ + *t*/*qe*) (3)

where *k*2 (min/mg) is the rate constant of the pseudo second order and *qe* is the amount adsorbed at equilibrium (mg/g). The plot of *t*/*qt* versus *t* gave a straight line for Cd and Cr with R^2^ values of 0.9895 and 0.9731, respectively. The experimental data were found to be in excellent agreement with the pseudo-second-order model (see Figure 10). The same results have been reported by [36]: the adsorption data for Cd and Cr were well fitted with the pseudo-second-order model. It is assumed that the adsorption mechanism follows chemisorption; the kinetics is fast; and the efficiency of each material is well suited to the model.

The intra-particle diffusion model is represented by the following equation:

*qt* = *k*_*p*_*t*^1/2^ + *C* (4)

where *kp* represents the intra-particle diffusion rate constant (mg/g min^1/2^) and intercept of plot *C* represents surface adsorption or the boundary effects. *C* is proportional to the thickness of the boundary layer; as the value of *C* increases, the boundary thickness also increases [37].

**Figure 11:**
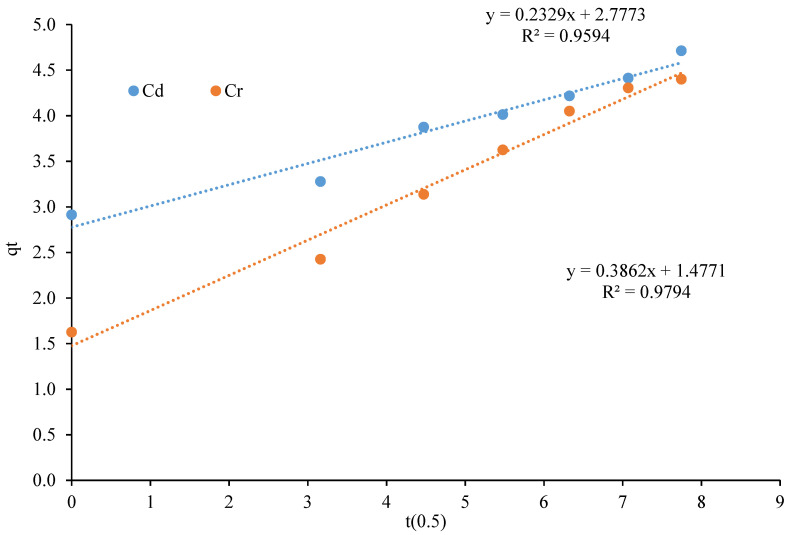
Intra-particle diffusion plot of metal adsorption on the CeO_2_/corncob nanocomposite.

Figure 11 shows the plot of *qt* vs. *t* ^0.5^. The values obtained for R^2^ were 0.9594 for Cd and 0.9794 for Cr, which suggests that the intra-particle diffusion model supports the adsorption of Cd and Cr. As the line did not pass through the origin, it shows that the intra-particle diffusion was not the only rate-controlling step, but other processes also involved the sorption process [38].

Figure 11 shows that the value of the intercept for Cd is 2.77 and Cr is 1.47, as [39] stated: when the value of *C*, is greater than 0, the intra-particle diffusion is not the rate-determining step in the adsorption process.

## 4. Conclusions

The removal of heavy metals from wastewater is a major concern of today and poses a great threat to human and environmental health. Therefore, this research study aimed to investigate the efficiency of a corncob nanocomposite for the removal of heavy metals from wastewater. For this study, the formation of a CeO_2_/corncob nanocomposite was investigated using FTIR, SEM, and XRD methods. The characterization results confirm the successful formation of the CeO_2_/corncob nanocomposite as observed by the clear change in spectra of FTIR, SEM, and XRD. The batch experiment for the adsorption of heavy metals revealed the successful removal, which is 95% for Cd and 88% for Cr. The adsorbent was synthesized via a simple approach, but it performed excellently towards the removal of Cd and Cr from wastewater. The results revealed that the CeO_2_/corncob nanocomposite is a remarkable adsorbent for the fast removal of Cd and Cr from wastewater. The adsorption capacity was influenced by pH, initial concentration, adsorbent dose, and contact time. The optimal removal for both Cd and Cr was at a basic pH. A pseudo-first-order kinetic model, pseudo-second-order kinetic model, and intra-particle diffusion model were used for the investigation of the experimental data. The data were in good agreement with the pseudo-second-order kinetic model and intra-particle diffusion model. A large amount of corncobs end up in landfill as waste; due to its abundance and availability, it can be used as an economic adsorbent for the removal of heavy metals from wastewater. Further studies to verify the efficiency of the CeO_2_/corncob nanocomposite for the removal of heavy metals in a large scale may be worthwhile to develop treatment strategies for industrial wastewater.

## Data Availability

The data presented in this study are available upon fair request from the corresponding author.
